# HIV-Specific T-Cells Accumulate in the Liver in HCV/HIV Co-Infection

**DOI:** 10.1371/journal.pone.0003454

**Published:** 2008-10-20

**Authors:** Bahareh Vali, Feng Yun Yue, R. Brad Jones, Prameet M. Sheth, Rupert Kaul, Michael R. Betts, David Wong, Colin Kovacs, Mona Loutfy, Andrew Common, Roberta Halpenny, Mario A. Ostrowski

**Affiliations:** 1 Institute of Medical Science, Department of Medicine, University of Toronto, Toronto, Canada; 2 Department of Immunology, University of Toronto, Toronto, Canada; 3 Department of Microbiology, University of Pennsylvania, Philadelphia, Pennsylvania, United States of America; 4 University Health Network, University of Toronto, Toronto, Canada; 5 Canadian Immunodeficiency Research Collaborative, Toronto, Canada; 6 St. Michael's Hospital, Toronto, Canada; University of California San Francisco, United States of America

## Abstract

**Background and Aims:**

Hepatitis C Virus (HCV)-related liver disease progresses more rapidly in individuals co-infected with Human Immunodeficiency Virus-1 (HIV), although the underlying immunologic mechanisms are unknown. We examined whether HIV-specific T-cells are identified in the liver of HCV/HIV co-infected individuals and promote liver inflammation through bystander immune responses.

**Methods:**

*Ex-vivo* intra-hepatic lymphocytes from HCV mono-infected and HCV/HIV co-infected individuals were assessed for immune responses to HIV and HCV antigens by polychromatic flow cytometry.

**Results:**

HCV/HIV liver biopsies had similar frequencies of lymphocytes but lower percentages of CD4^+^ T-cells compared to HCV biopsies. In co-infection, intra-hepatic HIV-specific CD8^+^ and CD4^+^ T-cells producing IFN-γ and TNF-α were detected and were comparable in frequency to those that were HCV-specific. In co-infected individuals, viral-specific CD8^+^ T-cells produced more of the fibrogenic cytokine, TNF-α. In both mono- and co-infected individuals, intra-hepatic HCV-specific T-cells were poorly functional compared to HIV-specific T-cells. In co-infection, HAART was not associated with a reconstitution of intra-hepatic CD4^+^ T-cells and was associated with reduction in both HIV and HCV-specific intra-hepatic cytokine responses.

**Conclusion:**

The accumulation of functional HIV-specific T-cells in the liver during HCV/HIV co-infection may represent a bystander role for HIV in inducing faster progression of liver disease.

## Introduction

Approximately 25% of Human Immunodeficiency Virus-1 (HIV) infected individuals are also infected with Hepatitis C Virus (HCV) [1]. HIV adversely affects each stage of the natural history of HCV infection. Fewer individuals recover spontaneously from HCV infection when also infected with HIV [2]. Among those with persistent HCV infection, HIV co-infection is associated with higher HCV viremia and more rapid progression to cirrhosis and hepatocellular carcinoma [3]. A recent meta-analysis showed that HIV co-infection increased the risk of histological hepatic cirrhosis by two-fold and clinically decompensated liver disease by six-fold [4]. In addition, HCV co-infection is associated with increased incidence of HAART (highly active antiretroviral therapy) related liver injury [5].

The mechanisms for hepatic damage in HCV/HIV co-infection are poorly defined. Although intra-hepatic T-cell immune responses are necessary for HCV clearance, they have also been shown to play a central role in mediating hepatocellular injury by direct cytotoxicity or indirectly by releasing cytokines. In this regard, IFN-γ has been shown to be anti-fibrogenic, whereas, TNF-α activates hepatic stellate cells, which induce fibrosis, and likely contributes to progression to cirrhosis [6], [7].

Potent and broad CD4^+^ and CD8^+^ T-cell immunity are important for virologic control in both HCV and HIV viral infections. *Ex-vivo* HCV-specific CD8^+^ T-cell responses in peripheral blood mono-nuclear cells (PBMCs) from mono-infected individuals are generally weak [8]. Although, peripheral HCV-specific CD4^+^ and CD8^+^ T-cell responses are somewhat weaker in HCV/HIV co-infected individuals [9], similar frequencies of intra-hepatic HCV-specific responses appear to be obtained in HCV versus HCV/HIV co-infection [10], [11]. However, *ex-vivo* HIV-specific CD8^+^ T-cell responses in PBMCs from HIV mono-infected individuals are about one log higher than *ex-vivo* HCV-specific responses in HCV mono-infection. In addition, impairment in cellular immune responses to HCV compared to HIV has been shown in HCV/HIV co-infection [12]. HIV-specific CD8^+^ T-cells are easily detectable in blood of untreated HIV infected individuals [13]. Such high frequencies of HIV-specific T-cells circulating in peripheral blood led us to question whether these cells could also migrate to the liver in HCV/HIV co-infection and through bystander responses add to the inflammation induced by HCV-specific T-cells.

## Materials and Methods

### Study participants

HCV mono-infected and HCV/HIV co-infected individuals who required liver biopsies for work up of liver disease were recruited for the study (see Results and Table 1). All study participants provided informed, written consent and the study protocol was approved by the research ethics board at the University of Toronto and St. Michael's Hospital. Both blood and liver biopsy samples were received from each participant.

**Table 1:** Characteristics of HCV mono-infected and HCV/HIV co-infected individuals, untreated for HCV.

	HCV mono-infected (n = 6)	HCV/HIV-1 Co-infected		p value
		not on HAART (n = 8)	on HAART (n = 12)	
Age (y, mean)	51(±3.5)	42(±5.7)	41(±2.8)	
Sex (no. male)	5	8	11	
HCV genotype (no. type 1)	5	7	11	
HCV plasma VL (log10 IU/ml, mean)	5.03±(0.51)*^a^	6.22(±0.12)*	6.3(±0.08)^a^	^a^*p<.05
HIV plasma VL (log10 copies/ml, mean)	na	6.5(±0.15)	1.43(±0.37)	p<.01
CD4 count/µl (mean)	na	492(±34.4)	599(±56.9)	p>.05
ALT (mean)	65(±13)*	70(±16)	109(±52)*	*p<.05
Liver Biopsy Inflammation Grade (mean)	1.7(±0.16)	1.9(±0.25)	1.7(±0.15)	p>.05
Liver Biopsy Fibrosis Score (mean)	3(±0.6)*	1.5(±0.3)*	2(±0.5)	*p<.05
Lymphocytes count/liver biopsy1 (mean)	43884(±11007)	47095(±7410)	59414(±5165)	p>.05

1. Normalized to 100,000 events count.

na = not applicabale.

no = number of participants.

HAART individuals were treated>1 year.

### Isolation of intra-hepatic lymphocytes from liver biopsy

Liver biopsy samples were washed in RPMI-1640 to remove contaminating blood lymphocytes, manually homogenized with a plastic plunger, and treated with DNase (0.002%, Sigma) and collagenase IV (0.02%, Sigma) for 30 minutes, stirring at 37°C. The digested cell suspension was filtered through a 70 µm strainer, washed and re-suspended in R-10 medium (10% fetal calf serum).

### IFN-γ ELISPOT epitope mapping in PBMCs

In order to identify candidate epitope-specific responses to be detected in *ex-vivo* liver samples, we first mapped antigen-specific T-cell responses in blood against the entire HIV-1 clade-B and HCV-1a proteome using the matrix approach by IFN-γ ELISPOT assay as described previously [14]. Mapped peptides were then pooled to evaluate hepatic responses. In order to address the possibility that differing epitopes were only targeted in the liver, we also used four peptide pools that previously were shown to target a majority of responses. These pools spanned HIV-Gag and HCV-NS3, HCV-NS4 and HCV-Core protein (2 µg/peptide/ml, from National Institute of Health Reagent Program). Of the HCV pools, the pool that gave the strongest ELISPOT response in PBMCs was used for hepatic cell stimulation (see below).

### *Ex-vivo* stimulation and intracellular staining

All the extracted cells from each liver biopsy were split in three wells and stimulated on the same day as PBMCs. 1×10^6^ PBMCs and liver- isolated cells were stimulated with either DMSO, HIV or HCV peptide pools as described previously[14]. HIV pools consisted of peptides that were screened by the matrix approach in that individual plus the HIV-Gag pool. Likewise, HCV pools consisted of mapped peptides plus an HCV pool that gave the strongest response in PBMCs. CD107a antibody (PE-Cy5, BD Pharmingen) was added at the time of stimulation. The following antibodies were used for staining: CD8-PE Texas-Red (Beckman Coulter), CD4-Pacific Blue (e-Bioscience), CD3- APCCY7, IFNγ-FITC, TNFα-PECY7, IL2-APC, MIP-1β-PE (BD Bioscience), PD-1 FITC (Biolegend) and dead cell stain Aqua (Invitrogen, Molecular Probes).

### Flow cytometry

Cells were analyzed on a multi-color FACSAria flow cytometer (BD Biosciences). For Blood samples between 500,000 to 1,000,000 total events and for liver biopsy samples between 50,000 to 200,000 total events were collected. Data analysis was performed using FlowJo version 8.6 (Treestar Inc., San Carlos, CA). Polychromatic FlowJo data were analyzed with PESTLE software, and pie-chart graphs were generated using SPICE software (obtained from M. Roederer, National Institutes of Health, Bethesda MD).

### Tetramer Staining

Multi-parameter analysis of HIV-Gag: 77–85 (SLYNTVATL: SL9) specific CD8^+^ T-cells was conducted in both blood and liver of co-infected individuals initially identified with a positive ELISPOT response to the 15-mer HIV peptide including SL9 epitope, using the corresponding tetramer (iTAg MHC Class-I tetramer, Beckman Coulter). Tetramer staining was performed prior to peptide stimulation, at room temperature for 20 minutes. Tetramer stained cells were then washed and stimulated with 10 µg/ml of SL9 peptide followed by ICS staining as mentioned above. Additional HIV, HCV and CMV-specific pentamer staining (Pro5 MHC class I Pentamers -Proimmune) was conducted followed by PD-1 staining.

### Statistical analysis

Data were analyzed by performing two-tailed non-parametric Mann-Whitney test using GraphPad Prism version 4.00. P-values≤0.05 were considered significant.

## Results

### Subject Characteristics

Three groups of individuals were studied as depicted in Table 1; HCV mono-infected (n = 6), HCV/HIV co-infected who were not receiving HAART (n = 8) and HCV/HIV co-infected who were receiving HAART for greater than one year at the time of evaluation (n = 12). All individuals never received prior treatment for HCV and underwent liver biopsies for staging and evaluation for pegylated-interferon/ribavirin treatment. HCV/HIV co-infected individuals had higher HCV viral loads. On average, CD4 T-cell counts of HIV infected individuals were >400/µl in both groups. Of note, the mean hepatic fibrosis scores were higher in the HAART treated and mono-infected groups in this cohort, indicating that individuals in these groups had more advanced disease at the time of biopsy in this study.

### Characterizing intra-hepatic lymphocytes

**Figure 1:**
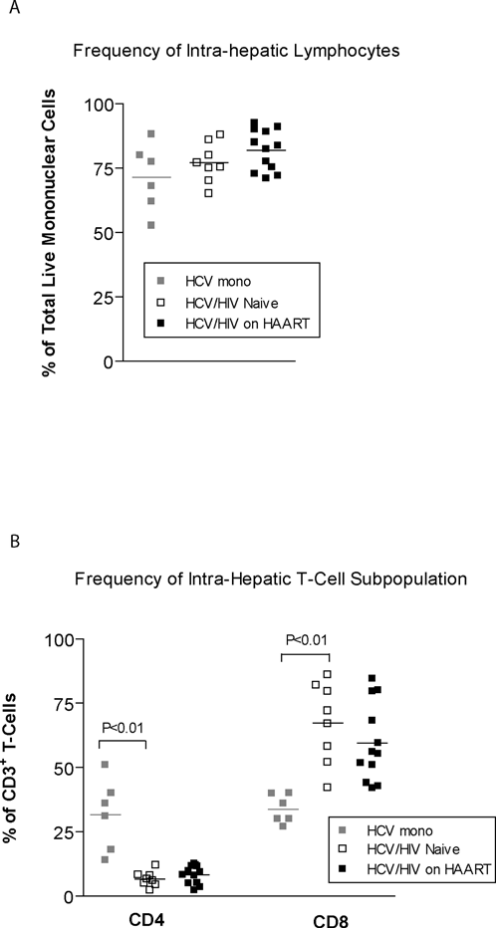
Characteristics of intra-hepatic lymphocytes from HCV and HCV/HIV co-infected individuals. Frequencies of lymphocytes obtained from liver biopsies normalized to total live mononuclear cell count from FACS analysis are illustrated in (a) with percent composition of intra-hepatic CD4 and CD8 expressing T-cells shown in (b).

Although similar frequencies of intra-hepatic lymphocytes were obtained in dual versus mono-infection, HAART-treated individuals showed a trend towards greater percentages of lymphocytes in their biopsies (Figure 1a). The percentage of intra-hepatic CD4^+^ T-cells was significantly reduced in dual infection [31.6%±13.8 for HCV vs 6.5%±2.9, for HCV/HIV therapy naïve, p<0.01], and was not associated with any improvement in HAART-treated individuals, as previously shown in the gut[15] (Figure 1b). However, compared to HCV mono-infected individuals the percentage of intra-hepatic CD8^+^ T-cells was higher in both co-infected groups [33.8%±5.5% for HCV vs 67.3%±15.5% for HCV/HIV therapy naïve vs 59.5±15.1% for HCV/HIV on HAART, p<0.01] (Figure 1b).

### Intra-hepatic viral specific immune responses

**Figure 2:**
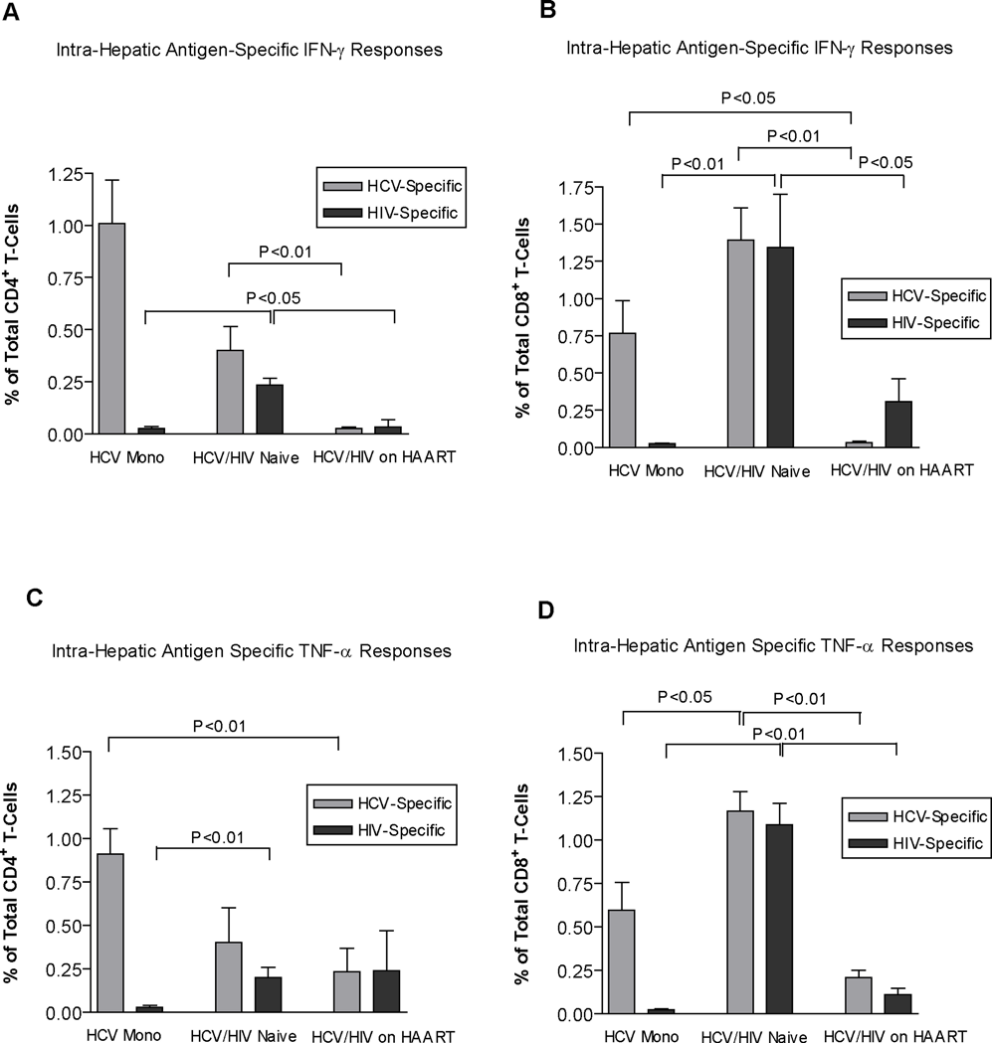
Antigen-specific cytokine production by intra-hepatic T-cells in HCV mono-infection and HCV/HIV co-infection. Figure (a) represents the summary data of the percentage of intra-hepatic CD4^+^ T-cells expressing IFN-γ in response to either HCV or HIV peptide stimulation in all the three studied cohorts, with figure (b) representing the same responses by CD8^+^ T-cells. Figures (c) and (d) represent the percentage of viral-specific, intra-hepatic TNF-α producing CD4^+^ and CD8^+^ T-cells respectively.

To determine the presence of intra-hepatic viral specific T-cell responses, liver isolated cells were stimulated with HCV and HIV peptide pools. Summary data of viral specific responses are depicted in Figure 2. In response to stimulation with HIV peptide pool, untreated co-infected individuals showed significantly higher frequencies of intra-hepatic CD4^+^ T-cells producing IFN-γ, compared to HCV mono-infected [0.16±0.05% vs 0.02±0.01%, p<0.05], and HAART-treated co-infected individuals [0.16±0.05% vs 0.03±0.05%, p<0.05] (Figure 2a). Untreated co-infected individuals showed a trend towards lower frequencies of intra-hepatic IFN-γ producing CD4^+^ T-cells in response to HCV peptides. Surprisingly, HAART-treated co-infected individuals had significantly reduced HCV-specific IFN-γ producing CD4^+^ T-cells when compared to untreated co-infected individuals [0.02±0.01% vs 0.46±0.11%, respectively, p<0.01] (figure 2a).

Therapy naïve co-infected subjects had greater IFN-γ producing CD8^+^ T-cells in response to HIV peptides compared to HCV mono-infected individuals [1.39±0.37% vs 0.02±0.0%, p<0.05], and HAART was associated with a significant reduction in the frequencies of these cells [1.39±0.37% vs 0.30±0.26%, p<0.05] (figure 2b). Although there was a trend for enhanced intra-hepatic CD8^+^ T-cells producing IFN-γ in response to HCV peptides in therapy-naive co-infection compared to HCV mono-infection, this was not found to be statistically significant. HAART on the other hand, was associated with a significant reduction in HCV-specific, intra-hepatic CD8^+^ T-cells producing IFN-γ [1.3±0.37% vs 0.03±0.01%, p<0.05] (figure 2b).

Similarly, co-infected individuals had significantly greater intra-hepatic TNF-α expressing CD4^+^ T-cells after HIV peptide stimulation compared to HCV mono-infected [0.2±0.05 vs 0.02±0.01, p<0.01], although HAART had no significant effect on their frequencies (Figure 2c). HCV mono-infected individuals showed significantly higher frequencies of HCV-specific TNF-α producing CD4^+^ T-cells compared to HAART-treated co-infected individuals [0.91±0.25% for HCV vs 0.23±0.20 for HCV/HIV on HAART, p<0.01] (figure 2c), but did not show significant differences with the untreated co-infected group.

The therapy-naïve co-infected group showed significantly higher frequencies of intra-hepatic TNF-α producing CD8^+^ T-cells in response to both HIV-1 and HCV antigens. Both types of responses were shown to be reduced in the HAART group [HIV-specific: 0.02±0.01% vs 1.08±0.21% vs 0.11±0.06%; HCV vs HCV/HIV vs HCV/HIV on HAART, p<0.01 for all], [HCV-specific: 0.59±0.2% vs 1.16±0.19% vs 0.20±0.07%; HCV vs HCV/HIV vs HCV/HIV on HAART, p<0.05 for all] (figure 2d).

### Multi-parameter analysis of T-cell functions

**Figure 3:**
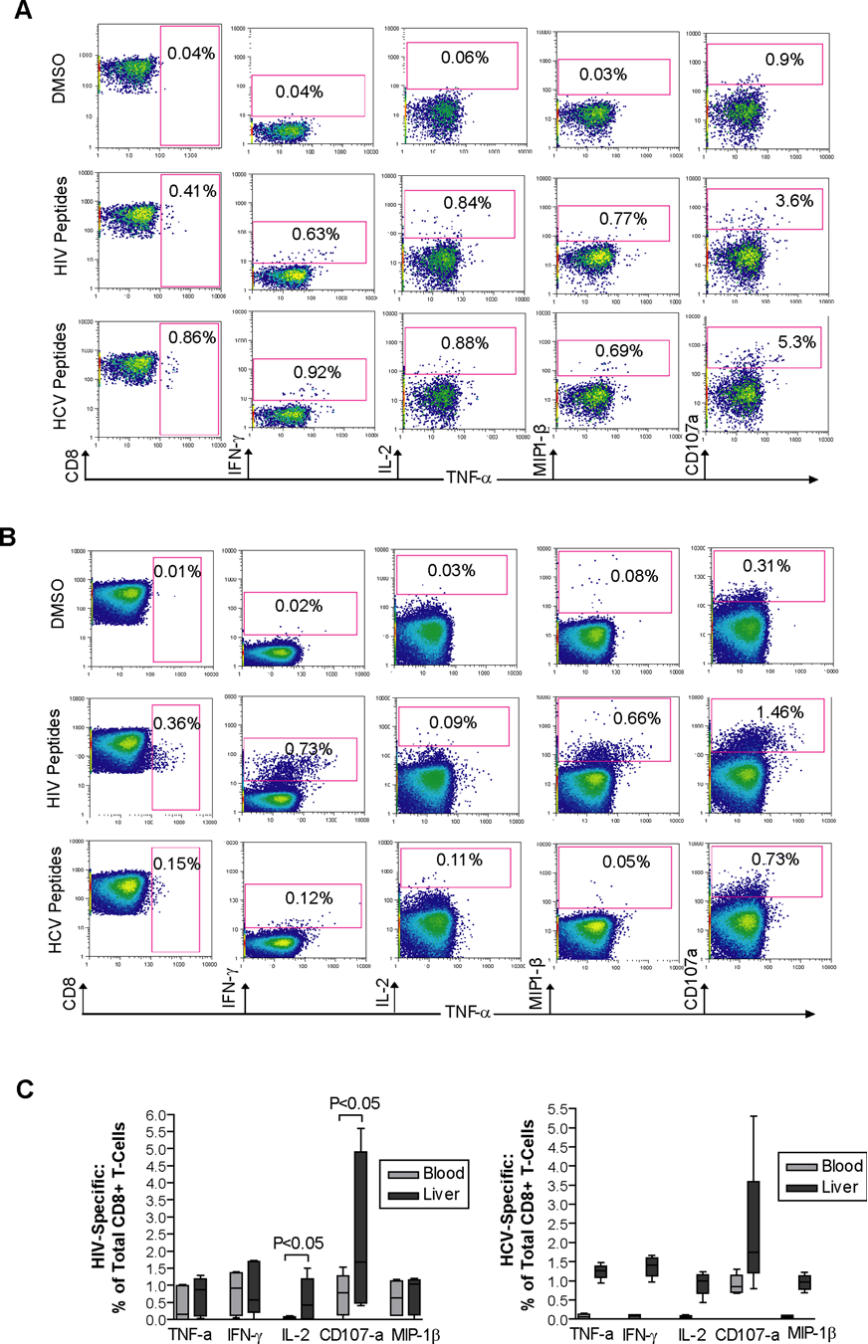
Polychromatic FACS analysis of viral-specific T cells in HCV/HIV co-infection. Shown are representative FACS data of the HIV and HCV specific multi-parameter CD8^+^ T-cell responses from (a) liver and (b) blood of subject OM 405, a therapy-naïve HCV/HIV co-infected individual, after in vitro stimulation using pool of HIV and HCV peptides. Initial gating on forward scatter area (FSC-A) versus height (FSC-H) was used to remove doublets. The events were further gated on forward scatter (FSC) versus the dead cell marker to remove dead cells. Lymphocytes were gated on the remaining live cells on a FSC versus SSC plot. Gates on CD3^+^/CD8^+^ cells were then generated. All responses are gated on a CD3^+^/CD8^+^ population and presented against TNF-α on the x-axis. Figure (c) shows a comparison of the frequency of HIV and HCV-specific CD8^+^ T-cells in the liver and blood of therapy-naïve, co-infected individuals. All intra-hepatic HCV-specific responses are significantly stronger than peripheral HCV-specific responses.

To study the functional profile of virus-specific T-cells in HCV/HIV co-infection, simultaneous expression of 5 distinct CD8^+^ T-cell markers were analyzed in 3 individuals within each cohort using a previously developed multicolor flow cytometry method[16]. Expression levels of degranulation marker CD107a and cytokines IFN-γ, TNF-α and IL-2, as well as the chemokine MIP-1β were simultaneously measured in response to HCV or HIV peptides in both blood and liver of each individual. Figure 3 depicts a representative multi-parameter analysis of CD8^+^ T-cell responses in liver and blood of a therapy-naïve, co-infected subject in response to HIV and HCV peptide pools. These data indicate that both HCV and HIV specific CD8^+^ T-cells expressing one or more functions are detectable in the liver and blood. Compared to blood, the frequency of HIV-specific T-cells producing CD107a and IL-2 was shown to be significantly higher in the liver of therapy-naïve, co-infected individuals. Consistent with previously reported data[10], HCV-specific responses were compartmentalized to the liver and stronger than peripheral HCV-specific responses (Figure 3c).

### Recognition of functional CTL, specific for the HLA-A0201-restricted HIV-SL9 epitope in HCV/HIV co-infected liver

**Figure 4:**
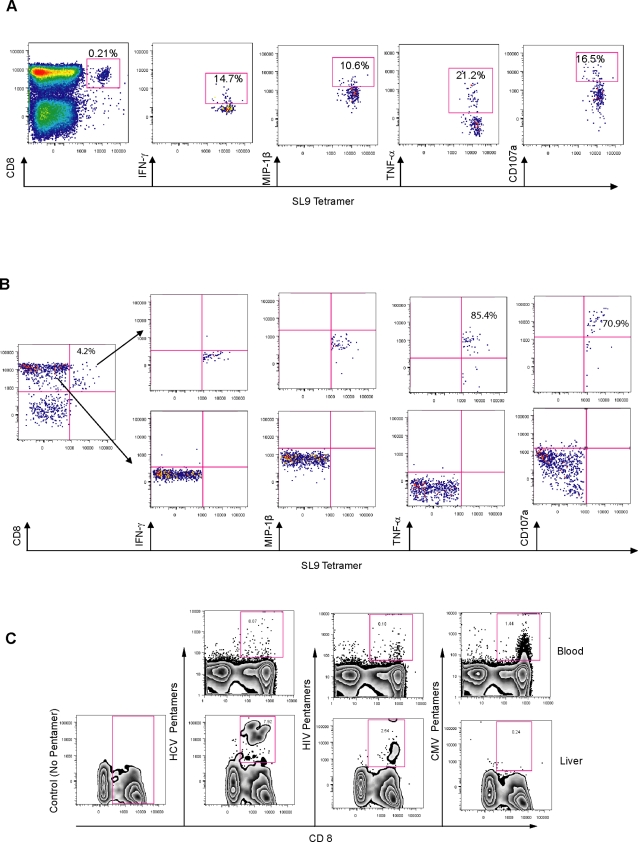
Tetramer positive cells specific for HIV are identified in the liver in HCV/HIV co-infection. Representative multi-parameter FACS plots of HIV-SLYNTVATL (SL9) tetramer positive/ CD8^+^ T-cells are shown in a) blood and b) liver of a co-infected individual (OM 403). Gating scheme on tetramer positive responses are based on initial gating on responses from the tetramer negative population. Figure (c) represents the frequency of CD8^+^ T-cells specific for HCV, HIV and CMV in HCV/HIV co-infected liver (OM 385). Liver isolated mononuclear cells were stained with pools of HLA-A*0201 and HLA-A*2402-restricted pentamers (Pro5 MHC class I Pentamers, Proimmune), followed by staining for cell surface markers CD3 and CD8. The following pentamers were used for each group: HCV pentamers: NS3-CINGVCWTV and NS3-KLVALGINAV; HIV pentamers: Pol-ILKEPVHGV and Gag p24-TLNAWVKVV; CMV Pentamers: pp65-NLVPMVATV and pp65-QYDPVAALF. No CD8^+^ T-cells specific for CMV were detected in this co-infected liver sample. Similar findings were found in another individual (data not shown).

To determine if T-cells specific for an HIV immuno-dominant epitope are present in HCV/HIV co-infected liver, we quantified CD8^+^ T-cells specific for HLA-A*0201-restricted SLYNTVATL (SL9) epitope in the liver of individuals with positive SL9 responses in their blood. We identified 3 co-infected HLA-A*0201 individuals, among them one showed a response to the SL9 epitope of HIV-Gag antigen. Figure 4 shows the multi-parameter analysis of tetramer positive CD8^+^ T-cells in blood and liver of this therapy-naïve, co-infected individual. The tetramer cytokine response pattern was shown to be different in the liver compared to blood of the same individual, with diminished intra-hepatic tetramer-specific IFN-γ responses and an increase in both CD107a and TNF-α responses, with the majority of SL9 tetramer positive cells expressing these two markers.

We also included CMV as a non-hepatotropic control virus in our liver analysis. Using a pool of HLA-A*0201- and HLA-A*2402-restricted, CMV-specific pentamers, we did not detect any CMV-specific CD8^+^ T-cells in HCV/HIV co-infected liver, although we could readily detect them in blood (Figure 4c).

### Hierarchy of viral-specific CD8^+^ T-cell function during HCV mono and HCV/HIV co-infection

**Figure 5:**
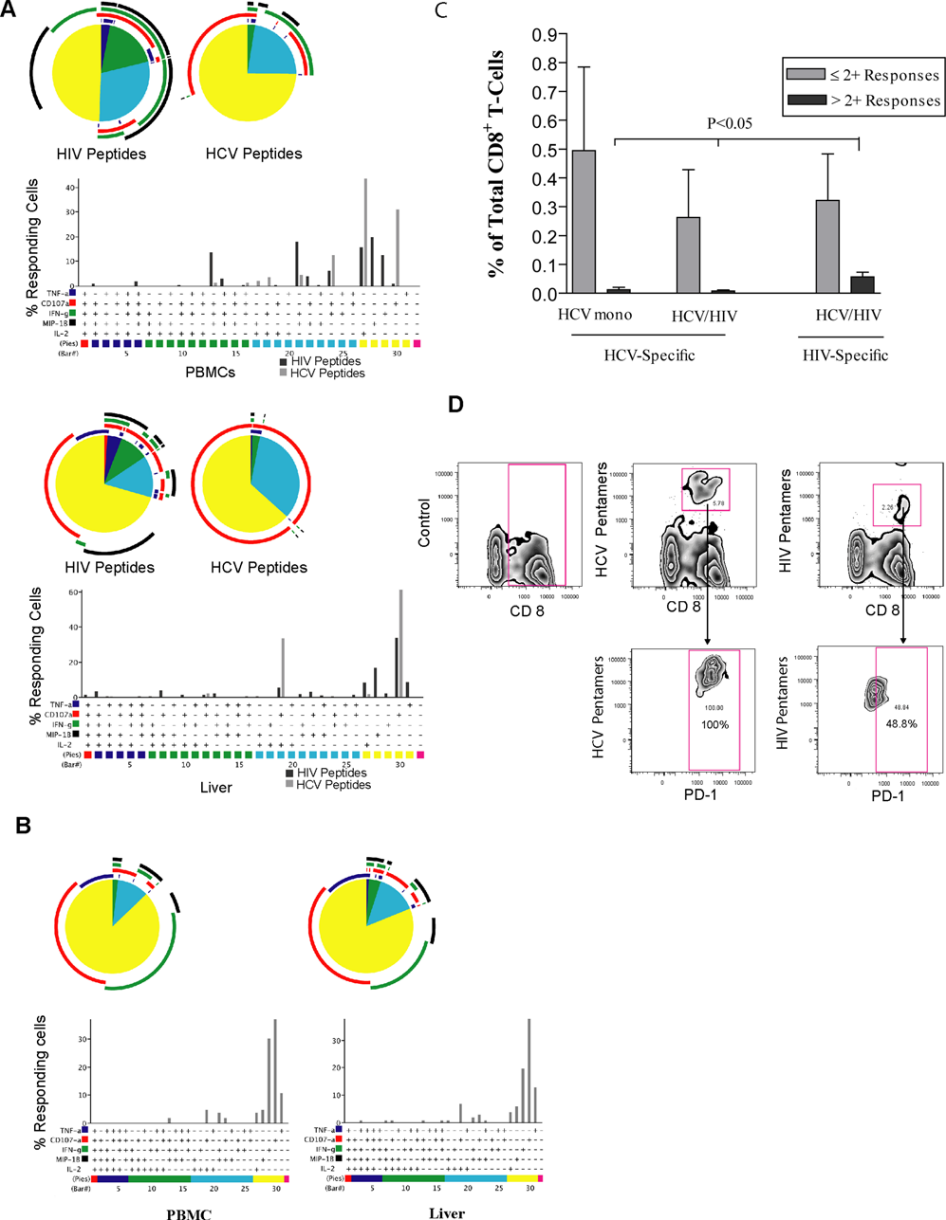
Characterization of the functional hierarchy of viral specific CD8^+^ T-cells in HCV/HIV co-infection. Representative functional profiles of virus specific CD8^+^ T-cells in blood and liver are depicted from an individual with (a) HCV/HIV co-infection (OM 405) and from an individual with (b) HCV mono-infection (OM 428). For multi-parameter analysis, the Boolean gating platform was used to create all of the possible combinations of functions, generating 32 response patterns for 5 of the different functions analyzed. All data are reported after background correction. Nonspecific background is shown to become extremely low when examining combinations of functions, nearly reaching 0 events for multiple functions simultaneously. This permits a very low threshold for detection of positive responses from multiple combinations. Consequently, for multi-parameter analysis, the results were thresholded based on a minimum criterion of positivity, as calculated by SPICE software and presented as the 90^th^ percentile of negative values for each analysis. Each pie chart generated by SPICE software, represents the hierarchy of responses to either HCV or HIV antigen stimulation. For simplicity, responses are grouped by number of functions and matched to the colored bars, with black bars representing the percentage of responding cells to HIV peptides and gray bars representing the percentage of responses to HCV peptide stimulation. In all pie charts, color red represents the 5+ responding population and the colors blue, green, turquoise, and yellow representing the 4+, 3+, 2+, and 1+ populations respectively. Color-coded arcs represent the dominant marker within each pie slice, with color blue representing TNF-α, red for CD107-a, green for IFN-γ and black for MIP-1β. Although IL-2 is included in the presentation and demonstrated by bar graphs, the software would not allow for arc colors for more than 4 responses. As a result there is no arc representative for IL-2. Figure (c) represents the average frequency of intra-hepatic viral specific responses within the pool of CD8^+^ T-cell populations simultaneously expressing 2 functions or less, compared with those within the pool of populations expressing more than 2 functions simultaneously; as analyzed in 3 subjects within each cohort of HCV mono and HCV/HIV co-infected individuals. The cutoff point of simultaneous expression of more than 2 measured markers is considered to show CD8^+^ T-cell poly-functionality. Figure (d) represents PD-1 levels on HIV-1 and HCV-specific T-cells from HCV/HIV Co-infected liver (OM 385). Liver cells were stained with two pools of HLA-A*0201-restricted pentamers (Proimmune): HCV pentamers: NS3-CINGVCWTV, NS3-KLVALGINAV and HIV pentamers: Pol-ILKEPVHGV, Gag p24-TLNAWVKVV. PD-1 gating was based on FMO (fluorescence minus one) of the control sample.

Using the panel of markers TNF-α, IFN-γ, MIP-1β, IL-2, and CD107a, we characterized the ability of CD8^+^ T-cells to simultaneously exert these ‘functions’ in response to both HCV and HIV peptides. Figure 5 depicts a representative functional profile of virus specific CD8^+^ T-cells in blood and liver during HCV/HIV co-infection (Fig. 5a) and HCV mono-infection (Fig. 5b). Analysis of co-infected subjects demonstrates a very limited functional hierarchy of HCV-specific T cells in the blood, with majority of T-cells producing one function. HIV-specific T-cells from blood had a more expanded functional hierarchy. In accordance with previous reports on HIV mono-infected individuals [16], cells expressing all 5 functions were absent in the blood of co-infected subjects, mainly due to lack of IL-2 production. In co-infection, intra-hepatic CD8^+^ T-cells responding to HCV peptides were within the single-responding and 2+ populations. Intra-hepatic CD8^+^ T-cell responses to HIV peptides produced a larger spectrum of responses. CD107a responding cells were represented in nearly all of the different HIV-specific populations in the liver of co-infected individuals.

As expected, HCV-specific responses in the blood of HCV mono-infected subjects were mainly single functional. The profile of HCV-specific responses in the liver of HCV mono-infected individuals showed the appearance of a very small population of 4+ responding cells in the liver.

We and others have considered a cutoff point of more than 2 simultaneously expressed markers to demonstrate poly-functional characteristic of responding T-cells [16], [17]. Figure 5c shows a comparison between average frequency of intra-hepatic viral-specific responses within the pool of CD8^+^ T-cell populations expressing 2 markers or less, and those within the pool of populations expressing more than 2 markers simultaneously. For both HCV and HIV-specific CD8^+^ T-cells the majority of responses had two or less functions. However, intra-hepatic HIV-specific responses demonstrated more poly-functionality, compared to HCV-specific responses either within co-infected or mono-infected individuals [0.05±0.01 vs 0.007±0.00, p<0.05; HIV-specific responses vs HCV-specific responses in HCV/HIV co-infected group]; [0.05±0.01 vs 0.01±0.00, p<0.05; HIV-specific responses in HCV/HIV co-infected group vs HCV-specific responses in HCV mono-infected group]. In summary, although viral-specific T-cells, simultaneously expressing all 5 measured markers were rarely found in the liver, intra-hepatic HIV-specific T-cells showed greater functional capacity when compared to those being HCV-specific.

Based on the recently highlighted role of PD-1 contributing to the dysfunction of T-cells in chronic viral infections, we also determined whether HIV and HCV-specific intra-hepatic T-cells differ in the degree of PD-1 expression. In an HCV/HIV co-infected liver, we found that 100% of intra-hepatic HCV-specific CD8^+^ T-cells were PD-1 positive, compared to 48.8% of those cells that were HIV-specific (Figure 5d).

## Discussion

This is the first study to demonstrate the presence of HIV-specific T-cells within the liver of HCV/HIV co-infected individuals. The finding of HIV-specific T-cells within liver of co-infected individuals may not altogether be surprising, given the high frequencies of HIV-specific CD8^+^ T-cells found in the peripheral blood in untreated HIV infection. Nevertheless, it is surprising to find functional T-cells of such viral specificities to be accumulating in liver. In contrast, we could not detect CMV-specific T-cells in co-infected liver despite their abundance in blood indicating that different viruses target T-cells to the liver. Recent studies have demonstrated that systemic viral infections may recruit viral-specific T-cells to the liver. The significance of non-hepatotropic viral-specific T-cells that are found in liver is unclear. It has been postulated that the liver can non-selectively trap activated T-cells during any infection, and thus act as a ‘sink’ or ‘graveyard’[18], however it is unclear whether these cells are rendered anergic while traveling in the liver or contribute to inflammation and damage as a result of bystander activation. Of note, is that hepatitis has been observed in measles [19], SARS [20] and in 20% of individuals with acute HIV infection [21]. Polakos et. al.[22] found that some individuals infected with influenza-A develop transaminitis and showed in a murine influenza model that influenza-specific CD8^+^ T-cells migrate to the liver and induce hepatic damage from by-stander activation. Non-hepatotropic viruses such as HIV, CMV and EBV, in general do not induce chronic hepatitis, thus, it is possible that the co-existence of hepatotropic viruses may alter the hepatic environment to allow recruitment of activated T-cells non-specifically. This could be due to an up-regulation of integrins such as ICAM-1 and VCAM-1 in hepatic sinusoids as previously shown during HCV infection [23] that could enhance T-cell recruitment. In this regard, Spangenberg et. al.[24] demonstrated the presence of influenza-specific T-cells in about 50% of liver biopsies from HCV mono-infected individuals.

There are several lines of evidence demonstrating that the liver efficiently clears many foreign pathogens, including RNA viruses. It is shown that liver is a major organ for clearing Simian Immunodeficiency Virus in rhesus monkeys [25]. There is also evidence for the detection of HIV RNA in the liver of HIV infected individuals [26]. These findings support the identification of HIV-specific T-cells in the liver. In HCV/HIV co-infection, it is possible that intra-hepatic HCV-specific CD4^+^ T-cells become infected with HIV and recruit HIV-specific immune responses to this site. Evidence for these potential mechanisms will need further analysis on liver biopsies of co-infected individuals.

Our analysis of liver biopsies from HCV/HIV co-infected individuals not only demonstrate that HIV-specific T-cells producing IFN-γ and TNF-α are detected in the liver, but also exhibit comparable frequencies of responses to those that are HCV-specific. This observation may explain the added contribution of HIV-specific immune responses to the ongoing intra-hepatic damage induced by HCV-specific T-cell responses that are inefficient in clearing the virus. Therapy naïve co-infected individuals demonstrated a higher frequency of intra-hepatic CD8^+^ T-cells that produce TNF-α in response to both HCV and HIV antigen stimulation compared to HCV mono-infected individuals. In addition, we identified CD8^+^ T-cells specific for an immunodominant HIV epitope in co-infected liver, demonstrating high frequency of TNF-α expression. Intra-hepatic TNF-α has been previously associated with liver fibrosis, and the accumulation of cells expressing this marker may explain in part the faster rate of liver disease progression found in HCV/HIV co-infection. Further comparisons of TNF-α responses between immunodominant HCV and other HIV epitopes in a larger cohort of individuals are warranted. Contrary to our expectation, viral-specific, intra-hepatic levels of IFN-γ were also higher in the therapy-naïve co-infected group, which would be against the expected protective role of IFN-γ. However, we interpret this observation as a potential effect of the fibrogenic TNF-α to mask IFN-γ protection. On the other hand, viral-specific T-cells are composed of several major populations with unique functional patterns. Therefore, measurement of only one or two T-cell functions may not provide a comprehensive picture of the quality of T-cell responses.

Recent lines of evidence demonstrate the importance of the qualitative rather than quantitative characteristics of CD8^+^ T-cell responses to efficient viral control [13], [27]. The significance of T-cell populations simultaneously representing 5 different functions has been discussed as a hierarchical functional model in viral infections such as CMV and EBV which are effectively controlled by respective CD8^+^ T-cells [16]. HCV-specific CD8^+^ T-cells were not poly-functional which is consistent with the notion that although HCV-specific T-cells are found in hepatic tissue, their loss of poly-functionality may be associated with inefficient control of HCV replication. HIV-specific T-cells in the liver of co-infected individuals however, simultaneously could express 4 and 5 of the measured markers. Recently, T-cell exhaustion has been related to signaling pathways through PD-1 [28], [29]. Our analysis of PD-1 levels of antigen-specific CD8^+^ T-cells from co-infected liver demonstrates higher expression of PD-1 on HCV-specific T-cells, compared to those specific for HIV, supporting the notion that the former are less functional. The observed poly-functionality of intra-hepatic HIV-specific T-cells, should have little effect on HCV replication but would further enhance the cytokine milieu induced from bystander activation, and contribute to liver damage during co-infection with HCV. In this regard, we found that the degranulation marker CD107a dominates the HIV-specific CD8^+^ T-cell responses in the liver, with the majority of the responding cells expressing CD107a, a surrogate marker for the cytotoxic function of CD8^+^ T-cells. Activated HIV-specific CD8^+^ T-cells with the potential to degranulate could induce bystander damage. In addition, the release of chemokines such as MIP-1β by the same cells could also attract further lymphocytes without HCV specificity to the liver. Bystander function of these non-specific T-cells could expand the tissue damage triggered by HCV infection and ultimately activate fibrogenesis.

We found that the frequency of CD4^+^ T-cells within livers of co-infected individuals was reduced compared to HCV mono-infection. Surprisingly, HAART did not appear to reconstitute the CD4^+^ T-cell population within liver. Despite this defect of CD4^+^ T-cell help, comparable frequencies of HCV-specific-CD8^+^ T-cells were found in co-infected livers. HAART-treated biopsies showed further reduced frequencies of HCV-specific responses. These data support previous findings that show HAART induces CD4^+^ T-cell recovery but not any restoration of HCV-specific T-cell responses peripherally [30]. Further investigation is needed to clarify the role of CD4^+^ T-cell help in affecting the frequencies of HCV-specific CD8^+^ T-cells in HCV/HIV-1 co-infection. HAART was also associated with a reduction in frequencies of HIV-specific T-cell responses within liver, indicating that removing the HIV antigenic load may also reduce the opportunity for such cells to accumulate within hepatic tissue.

Here, we propose a novel mechanism for enhanced HCV-related liver disease progression in HIV co-infection; that of bystander activation and induced inflammation from HIV-specific T-cells accumulated in the liver. Our data however are limited in the cross-sectional nature of our cohort, the low number of analyzed liver biopsies and the narrow range of CD4^+^ T-cell counts among the studied individuals. We should also acknowledge that *ex-vivo* functional T-cell capacity may not exactly reflect the situation *in-vivo*. Further studies, particularly, those which are prospective are warranted in order to understand the role that HIV-specific T-cells play in contributing to fibrosis and in particular how HAART modulates these responses.
